# Endogenous arginine vasopressin–positive retinal cells in arginine vasopressin–eGFP transgenic rats identified by immunohistochemistry and reverse transcriptase–polymerase chain reaction

**Published:** 2011-12-15

**Authors:** Satoru Moritoh, Kaori Sato, Yasunobu Okada, Amane Koizumi

**Affiliations:** 1 Department of Cell Physiology, National Institute for Physiological Sciences, Okazaki, Japan; 2 Section of Communications and Public Liaison, National Institute for Physiological Sciences, Okazaki, Japan; 3 Department of Physiological Sciences, School of Life Science, The Graduate University for Advanced Studies (SOKENDAI), Okazaki, Japan

## Abstract

**Purpose:**

Recently, arginine vasopressin (AVP) has been revealed to have diverse functional roles in nervous tissues beyond that of a vasoconstrictor. Several previous studies have indicated the existence of AVP in the retina, but the source of AVP has not been determined. The objective of the present study was to address the question of whether retinal cells have the ability to synthesize endogenous AVP to act in a paracrine or autocrine manner.

**Methods:**

We used AVP-eGFP transgenic rats to find endogenous AVP-positive cells in the retina by immunohistochemistry with an AVP antibody and a GFP antibody. We also examined *AVP* mRNA and AVP receptor genes by reverse transcriptase (RT)–PCR of dissociated GFP-positive cells and whole retinas.

**Results:**

Endogenous AVP-positive cells were found in the ganglion cell layer and inner nuclear layer of the retina of AVP-eGFP transgenic rats by immunohistochemistry. As indicated by the results of RT–PCR of dissociated GFP-positive cells, these cells have the ability to synthesize endogenous AVP, as well as transgenic AVP-eGFP. In addition, the V1a and V1b AVP receptors were found in the wild-type rat retina by whole retina RT–PCR, but the V2 receptor was not detectable. In dissociated AVP-eGFP-positive cells, no AVP receptor was detected by RT–PCR. Moreover, AVP secretion was not detected by stimulation with a high potassium (50 mM) solution.

**Conclusions:**

In the rat retina, we found retinal cells that have the ability to synthesize endogenous AVP, and that the retina possesses V1a and V1b AVP receptors. Taken together, these results suggest that the retina has an intrinsic AVP-synthesizing and -receiving mechanism that can operate in a paracrine manner via V1a and V1b receptors.

## Introduction

Arginine vasopressin (AVP) is a neuropeptide hormone released from the dorsomedial suprachiasmatic nucleus to regulate the homeostasis of osmolarity and the volume of body fluids [1]. AVP exerts its physiological effects through the V1a, V1b, and V2 receptors [2]; it is a potent stimulator of vascular smooth muscle contraction through V1a receptors, with a specific intracellular second messenger system [3]. AVP has an important role in the maintenance of cardiovascular homeostasis through these distinct receptors, which are potent therapeutic targets for the treatment of heart failure and the regulation of blood pressure [4,5].

Recently, roles of AVP other than its role as a vasoconstrictor have been revealed. AVP-positive cells were discovered in the olfactory bulb, and were shown to be related to olfactory function and social recognition rather than vasoconstriction [6]. Vasopressin is now known to be a key factor of social recognition in the brain [7]. In fact, AVP-receptor V1a knockout mice showed impairment of social recognition [8]. In the hypothalamus of the rat brain, AVP regulates the cell volume of AVP-positive cells in an autocrine manner [9].

Although several studies have indicated the presence of AVP in the retina [10-12], little is known about the source and function of retinal AVP. Djeridane [10] reported that AVP was detected by immunohistochemistry in the retinal ganglion cell layer (GCL), although Djeridane suggested that AVP itself is not synthesized in retinal cells. Palm et al. [12] reported that AVP was clearly present in the eye, but that it might be stored after accumulation from blood or cerebrospinal fluid, or possibly produced locally. In regard to the function in the eye, AVP was primarily thought to have vasoactive/vascular effects on the endothelium to regulate blood flow [13,14]. In addition to its vasoactive/vascular effects, AVP may have a pathological role in regulating intraocular pressure via the vasopressin V1 receptor [15]. It was also reported that the human retinal pigment epithelium in culture possesses the vasopressin V1 receptor [16]. The remaining questions are whether AVP itself is synthesized in the retina or whether it just comes from extraretinal brain tissue through blood vessels, as well as whether AVP acts on the retina in a paracrine or autocrine manner.

The objective of the present study was to address the question of whether retinal cells have the ability to synthesize endogenous AVP. To answer this question, we examined AVP-enhanced green fluorescent protein (eGFP) transgenic rats to find endogenous AVP-positive retinal cells by immunohistochemistry with an AVP antibody and a GFP antibody, and by reverse transcriptase (RT)–PCR. AVP-eGFP transgenic rats were designed to express AVP-eGFP in AVP-secreting cells under the control of the *AVP* promoter [17]. We found that there were AVP-positive cells in both the inner nuclear layer (INL) and GCL of the rat retina. In addition, the V1a and V1b AVP receptors were found in the retina. Although the level of endogenous AVP expression was quite low, we suggest that the retina has an intrinsic AVP-synthesizing and -receiving mechanism that can operate in a paracrine manner.

## Methods

### The arginine vasopressin–eGFP transgenic rat

Wild-type male Wistar rats (Charles River Laboratories, Yokohama, Japan) and heterozygous transgenic male Wistar rats that express an AVP-eGFP fusion gene [17] were bred and housed under standardized conditions (12 h:12 h light-dark cycle) with food and water. Five- to 12-week-old wild-type and AVP-eGFP transgenic rats were used for experiments. Light-adapted rats were deeply anesthetized with halothane (Takeda Pharmaceutical, Osaka, Japan) and sodium pentobarbital (Dainippon Pharmaceutical, Osaka, Japan), and then sacrificed by decapitation. Eyes were removed, transferred to carboxygenated Ames’ medium (Sigma-Aldrich, St. Louis, MO), and hemisected, and then the retinas were teased off the pigment epithelium. All procedures involving animals were approved in advance by the Ethics Review Committee for Animal Experimentation of the National Institute for Physiological Sciences, and were in accordance with the guidelines of the Physiological Society of Japan.

### Immunostaining

The isolated retinas were fixed in phosphate buffered saline (PBS), pH 7.4, containing 4% paraformaldehyde for 1 h at room temperature. The retinal preparations were rinsed with PBS and incubated for 1 h at 4 °C in PBS with 4% donkey serum (Biowest, Nuaillé, France) and 0.3% Triton X-100 (Katayama Chemical, Osaka, Japan) for blocking before the addition of primary antibodies. Primary antibodies against AVP (1:800, rabbit polyclonal; Calbiochem, Darmstadt, Germany) and GFP (1:500, goat polyclonal; Rockland Immunochemicals, Gilbertsville, PA) were used. The preparations were incubated in primary antibody solution for at least 4 days at 4 °C (whole mounts). After several rinses in PBS, preparations were incubated with secondary antibodies (donkey antirabbit IgG coupled to Alexa Fluor 594 or donkey antigoat IgG coupled to Alexa Fluor 488, from Invitrogen) diluted 1:1,000 in blocking buffer overnight at 4 °C, rinsed, and coverslipped with Fluoromount-G (SouthernBiotech, Birmingham, AL). Images were captured using confocal laser scanning microscopy (A1R; Nikon). Nikon PlanApo VC20X and 40X lenses were used. The brightness and contrast of the images were adjusted by using ImageJ (National Institutes of Health, Bethesda, MD) or Photoshop CS (Adobe, San Jose, CA).

### Density recovery profile

**Figure 1:**
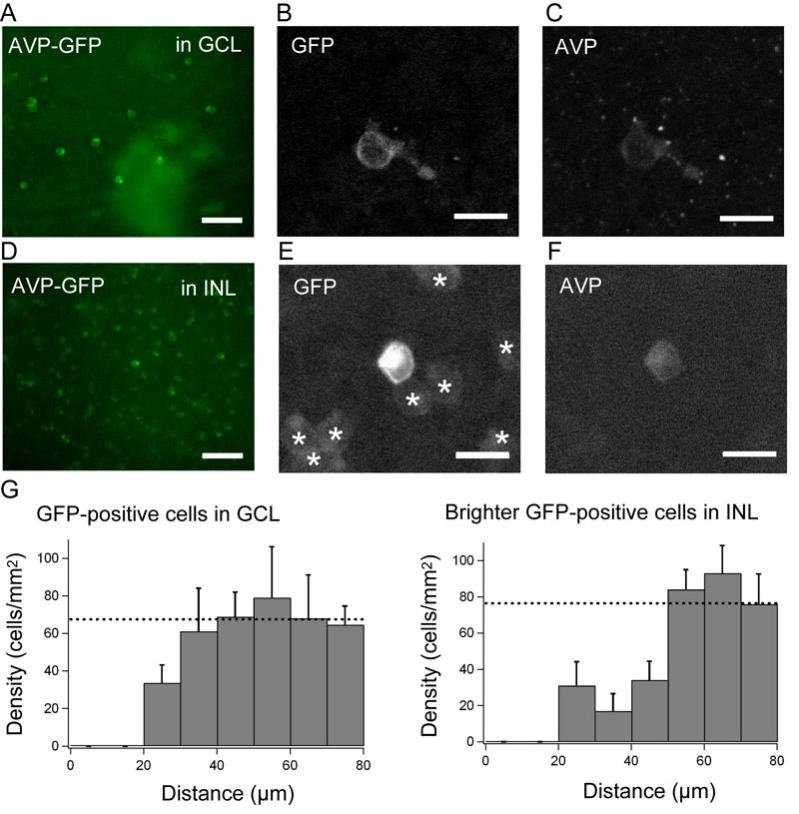
Endogenous arginine vasopressin–positive cells in the arginine vasopressin–eGFP transgenic rat retina. Arginine vasopressin (AVP)/eGFP-positive cells in (**A-C**) the ganglion cell layer (GCL) and (**D-F**) inner nuclear layer (INL) of the AVP-eGFP transgenic rat retina in a whole-mount preparation. **A**: AVP-eGFP expression in the GCL under a fluorescence microscope. **B**: A cell with GFP in the GCL detected by a confocal microscope. **C**: The GFP-positive cell in **B** was also positive for AVP. **D**: AVP-eGFP expression in the INL under a fluorescence microscope. **E**: Cells with GFP in the INL, detected by a confocal microscope. There are two distinct types of GFP-positive cells: brighter cells and faint cells. Asterisks show faint cells. **F**: The brighter GFP-positive cell in **E** was also positive for AVP. The scale bars in **A** and **D** are 50 μm. The scale bars in **B**, **C**, **E**, **and F** are 10 μm. **G**: Density recovery profile (DRP) of GFP-positive cells in the GCL and brighter GFP-positive cells in the INL (n=7 retina pieces). Error bars indicate standard error of the mean (SEM).

The spacing of cell bodies of AVP-eGFP-positive cells in the INL and the GCL (Figure 1) was analyzed using the density recovery profile (DRP) method, calculated in MatLab (The Mathworks, Lowell, MA) [18]. The concept of DRP analysis used the spatial autocorrelogram, as described previously [19].

### Dissociation of GFP-positive cells from the arginine vasopressin–eGFP transgenic rat retina

Small pieces of retinas were cut off and dissociated in oxygenized 5 mM HEPES Hank’s balanced salt solution (HBSS; Invitrogen, Carlsbad, CA), pH 7.4, containing 15.6 U/ml papain (Worthington, Lakewood, NJ) and 12.5 μg/ml L-cysteine (Sigma-Aldrich) for 10 min at 37 °C. The retina pieces were washed once with 5 mM HEPES HBSS containing 200 U/ml DNase I (Sigma-Aldrich) and four times with 5 mM HEPES HBSS, and then stored in 5 mM HEPES HBSS bubbled with 100% O_2_ at room temperature. The cells were dissociated by mechanical trituration through a Pasteur pipette. GFP expression was confirmed each time under a fluorescence microscope (TE300; Nikon, Tokyo, Japan).

### Reverse transcriptase–polymerase chain reaction from GFP-positive cells

**Figure 2:**
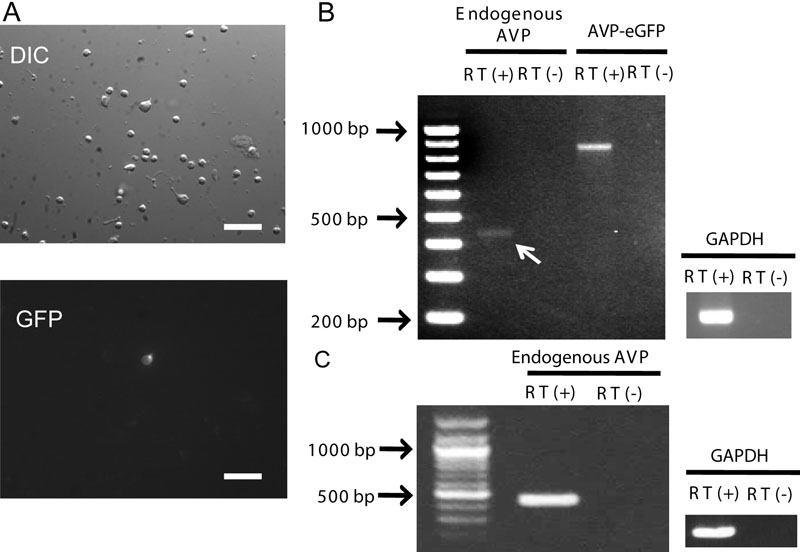
Endogenous arginine vasopressin mRNA was detected by reverse transcriptase–polymerase chain reaction from dissociated GFP-positive cells and the whole retina. **A**: Dissociation of the arginine vasopressin (AVP)-eGFP transgenic rat retina. Under a fluorescence microscope, we identified AVP-eGFP-positive cells by GFP fluorescence. Scale bars are 20 μm. **B**: Reverse transcriptase (RT)–PCR from a set of 20 dissociated GFP-positive cells. A faint endogenous AVP band (white arrow) and an AVP-eGFP band were detected by the indicated primer pairs (Table 1) after 45 cycles of RT–PCR. **C**: RT–PCR from the wild-type whole retina. An endogenous *AVP* band was also detected.

**Figure 3:**
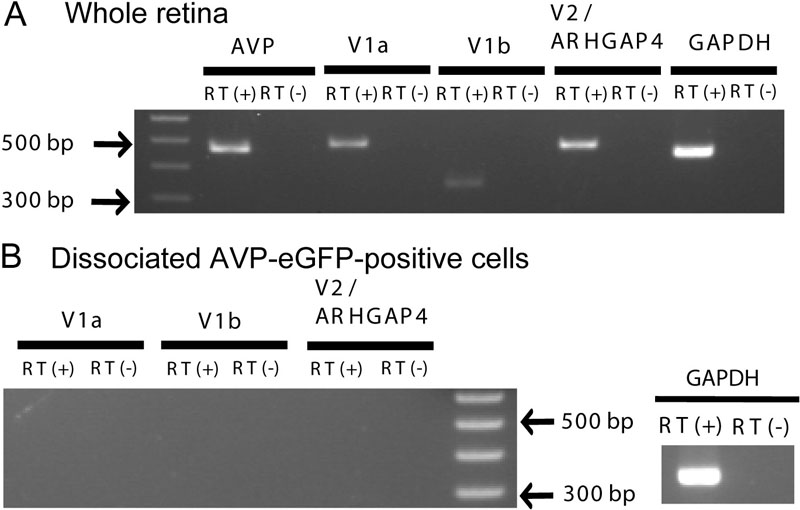
Arginine vasopressin receptors in the retina detected by whole-retina reverse transcriptase–polymerase chain reaction. **A**: Arginine vasopressin (AVP) receptors were detected from a whole wild-type rat retina by reverse transcriptase (RT)–PCR with the indicated primer pairs (Table 1). V1a and V1b receptors were detected. A V2/*ARHGAP4* band was also detected, but by cloning and sequencing the RT–PCR product, we concluded that the V2/*ARHGAP4* was from the *ARHGAP4* gene, which overlaps the V2 receptor gene and is expressed from the opposite DNA strand of the V2 receptor gene. **B**: No AVP receptor was detected by RT–PCR in dissociated AVP-eGFP-positive cells, as shown in Figure 2B.

The techniques used were identical to those previously described [9,20]. Using a RNeasy Micro Kit (Qiagen, Hilden, Germany), we extracted total cellular RNA from cytosol suctioned into gigasealed patch pipettes from 20 eGFP-positive cells and pooled them. RNA samples were reverse-transcribed with random 6-mer primers using a PrimeScript 1st strand cDNA Synthesis kit (Takara Bio, Otsu, Japan) according to the manufacturer’s protocol. PCR was performed with 1 U of Blend Taq DNA polymerase (Toyobo, Osaka, Japan) in a 40 μl reaction mix contained 1× buffer, 0.2 mM deoxynucleotide triphosphates (dNTPs), and 3 μl cDNA template. Primer sequences are shown in Table 1. Note that we used two different pairs of primers to detect endogenous *AVP* in experiments for which the results are shown in Figure 2 and Figure 3. Amplification was performed in a thermal cycler (GeneAmp PCR System 9700; Applied Biosystems, Foster City, CA) under the following conditions: initial heating at 94 °C for 2 min followed by 45 cycles of denaturation at 94 °C for 30 s, annealing at 60 °C for 30 s, extension at 72 °C for 1 min, and then final extension at 72 °C for 5 min for endogenous *AVP* and transgene products of AVP-eGFP; and initial heating at 94 °C for 2 min followed by 35 cycles of denaturation at 94 °C for 30 s, annealing at 60 °C for 30 s, extension at 72 °C for 30 s, and then final extension at 72 °C for 5 min for glyceraldehyde-3-phosphate dehydrogenase (*GAPDH*) as a positive control.

**Table 1:** RT–PCR primer sequences.

**mRNA species**	**Sequence**	**RT–PCR product (bp)**	**Accession number**	**Reference^1^**
**Endogenous AVP (in****Figure 2****)**				
AVP-F	CCTCACCTCTGCCTGCTACTT	430	NM_016992.2	
AVP-R2	AGAATCCACGGACTCTTGTGT			
**AVP-eGFP**				
AVP-F	CCTCACCTCTGCCTGCTACTT	843		
AVP-eGFP-R	ATGATATAGACGTTGTGGCTGTTGT			
**GAPDH**				
GAPDH-F	CATGCCGCCTGGAGAAACCTGCCA	429	NM_017008.3	[9]
GAPDH-R	GGGCTCCCCAGGCCCCTCCTGTTG			
**AVP (in****Figure 3****)**				
AVP-F	CCTCACCTCTGCCTGCTACTT	463	NM_016992.2	[21]
AVP-R	GGGGGCGATGGCTCAGTAGAC			
**V1a receptor**				
V1a-F	CGCAACATCCGCGGAAAGAC	484	NM_053019.2	[9]
V1a-R	TCCACATCCCAGTGCTGTTC			
**V1b receptor**				
V1b F	CAGCCAGTCTACCTACCCTCTCA	335	NM_017205.2	
V1b R	CCCTCGTGGCTGCAGAAGAT			
**V2 receptor/ARHGAP4^2^**				
V2/ARHGAP4-F	CGTGGGATCCGGAAGCTCCTCTGG	301 (V2 receptor)	NM_019136.1	[22]
V2/ARHGAP4-R	TCAGGGCCAACCCTAGATAGTCAG	462 (ARHGAP4)	AY742900.1	

^1^Published paper that RT–PCR primers have been already designed and used. ^2^*ARHGAP4* gene overlaps V2 receptor gene and is expressed from the opposite DNA strand of the V2 receptor gene in rat tissues [24].

### Whole-retina reverse transcriptase–polymerase chain reaction

Using TRIzol Reagent (Invitrogen), total RNA was extracted from the retinas of six-week-old male Wistar rats (Charles River Laboratories). RNA samples were treated with Recombinant DNase I (RNase-free; Takara Bio) and reverse-transcribed with oligo-dT primers using Superscript III RT (Invitrogen) according to the manufacturer’s instructions. *AVP* PCR primers designed by Glasgow et al. [21], V1a and *GAPDH* PCR primers designed by Sato et al. [9], and V2/a Rho GTPase-activating protein 4 (*ARHGAP4*) PCR primers designed by Vargas et al. [22] were employed. V1b PCR primers were designed with Primer3 software. The sequences of the primers are listed in Table 1. PCR was performed with Blend Taq DNA polymerase (Toyobo) under the following conditions: initial heating at 94 °C for 2 min followed by 25 cycles (*GAPDH*), 35 cycles (*AVP*, V1a, and V2/*ARHGAP4*), or 40 cycles (V1b) of denaturation at 94 °C for 30 s, annealing at 60 °C for 30 s, extension at 72 °C for 30 s, and then final extension at 72 °C for 5 min. The products of RT–PCR were electrophoresed on a 2% agarose gel, the bands were excised, and then the products were cloned into the pGEM-T Easy vector (Promega, Madison, WI) after purification with the Wizard SV Gel and PCR Clean-Up System (Promega). Plasmids were extracted with PI-200 (Kurabo, Osaka, Japan), treated with RNase (DNase free) Glycerol Solution (Nippon Gene, Tokyo, Japan), purified by phenol-chloroform extraction and ethanol precipitation, and used as templates for sequencing using a Big-Dye Terminator v3.1 Cycle Sequencing Kit (Applied Biosystems) and an ABI PRISM 310 Genetic Analyzer (Applied Biosystems).

### Measurements of arginine vasopressin secretion

The amount of AVP secretion from whole retinas or dissociated retinal cells exposed to control solution or high potassium (50 mM) solution was measured at room temperature using an arg^8^-Vasopressin Enzyme Immunoassay Kit (Assay Designs, Ann Arbor, MI) according to the manufacturer’s protocol. The control solution contained (in mM): 125 NaCl, 2.5 KCl, 2 CaCl_2_, 1 MgCl_2_, 26 NaHCO_3_, 1.25 KH_2_PO_4_, and 12 glucose (pH 7.4; 300 mosmol/kg-H_2_O). The high potassium (50 mM) solution contained (in mM): 77.5 NaCl, 50 KCl, 2 CaCl_2_, 1 MgCl_2_, 26 NaHCO_3_, 1.25 KH_2_PO_4_, and 12 glucose (pH 7.4; 300 mosmol/kg-H_2_O).

For the measurements, whole retinas were isolated from four 5-week-old male wild-type Wistar rats (Charles River Laboratories). Then whole retinas were put in a carboxygenated custom-made chamber. After 1.5 h of preincubation in the control solution, retinas were rinsed once in the control solution and then each retina was incubated for 15 or 30 min in 5 ml control solution or high potassium (50 mM) solution. Each time, 0.5 ml was sampled for measurements.

Dissociation of retinal cells was performed using two retinas of a two-month-old wild-type Wistar rat (Charles River Laboratories), similar to the procedure for the dissociation of the AVP-eGFP transgenic rat retina in this study. After a 20 min preincubation of a batch of dissociated cells, the cells were incubated for 5, 15, or 30 min in the control solution or the high potassium (50 mM) solution. These solutions were then centrifuged at 500× g for 5 min at 4 °C and the supernatants were used for measurements.

Extraction of AVP was performed by application onto a C-18 SEP-COLUMN (Phoenix Pharmaceuticals, Burlingame, CA) according to the manufacturer’s protocol using Buffers A and B (Phoenix Pharmaceuticals). Extracted AVP was dried with a vacuum freeze dryer (VD-800F; Taitec, Koshigaya, Japan). The amount of AVP was estimated with a Microplate reader (Multiscan MS-UV; Labsystems, Vienna, VA) at a wavelength of 414 nm. The concentration of AVP was estimated each time under the condition that the concentration was set to 0 in the solution before incubation of whole retinas.

## Results

### Endogenous arginine vasopressin–positive cells in the arginine vasopressin–eGFP transgenic rat retina

The AVP-eGFP transgenic rat was designed to express AVP-eGFP to recapitulate the expression of endogenous *AVP* in AVP-secreting cells under control of the *AVP* promoter [17]. In the transgenic rat, the AVP-eGFP fusion gene was expressed under the *AVP* promoter in cells that had endogenous *AVP* promoter activity [6,17]. In the retina of the AVP-eGFP transgenic rat, AVP-eGFP-positive cells were identified in the retinal GCL and the INL by immunoreactivity to the GFP antibody (Figure 1A,D). All of the GFP-positive cells in the GCL were also immunoreactive to the AVP antibody (Figure 1B,C), although only 5% of the GFP-positive cells in the INL were costained with the AVP antibody (Figure 1E,F). In the INL, there were two distinct types of GFP-positive cells: brighter cells and faint cells (Figure 1E, asterisks on faint cells). Almost all of the brighter GFP-positive cells in the INL were costained with an AVP antibody, although faint cells were not. The number of AVP-eGFP-positive cells in the GCL was 67.6 ±12.5 cells/mm^2^ (mean±standard error of the mean [SEM], n=7 retina pieces). In the INL, the number of brighter AVP-eGFP-positive cells was 76.4±7.0 cells/mm^2^ and the number of faint GFP-positive cells was 1,500±84 cells/mm^2^. The DRP of GFP-positive cells in the GCL and brighter cells in the INL showed a mosaic distribution with a space size of ~50 μm (Figure 1G).

AVP-eGFP-positive cells in the GCL had no GFP-positive neurites/axons in the optical nerve layer identified by GFP immunoreactivity. In addition, the diameter of the soma of AVP-eGFP-positive cells in GCL was about 8 μm, similar to that of the brighter cells in the INL. Although some retinal ganglion cells can have somas of only ~11 μm diameter [23], small soma size is another good indication that these are a subtype of amacrine cells. Taken together, the results indicate that brighter AVP-eGFP-positive cells in both the GCL and INL are probably a subtype of amacrine cells with endogenous AVP.

### Endogenous arginine vasopressin mRNA in dissociated GFP-positive cells

The next question is whether AVP-eGFP-positive cells have the ability to synthesize endogenous AVP. We assessed the endogenous AVP–synthesizing ability by RT–PCR from dissociated GFP-positive cells (Figure 2). We dissociated the transgenic rat retina enzymatically and collected the cytosol from brighter GFP-positive cells under a fluorescence microscope using Microglass pipettes (see Methods) [9]. RT–PCR was conducted to detect the transgene mRNA of AVP-eGFP and the endogenous *AVP* mRNA with primer pairs shown in Table 1 from a set of 20 GFP-positive cells [9,20]. After 45 cycles of RT–PCR, we found an endogenous *AVP* mRNA band, as well as a transgenic AVP-eGFP mRNA band (Figure 2B). To confirm that the wild-type retina has the ability to synthesize AVP, we checked the *AVP* mRNA expression in the wild-type whole retina. An endogenous *AVP* band was also detected in the wild-type retina (Figure 2C). Overall, AVP-eGFP-positive retinal cells have the ability to synthesize endogenous AVP.

### Arginine vasopressin receptors in the whole retina

Next, we examined the possibility that AVP acts on the retina in a paracrine or autocrine manner. If the AVP peptide itself is synthesized and released from the retina to act on retinal cells, there should be AVP receptors in the retina. We examined the *AVP* receptor mRNA from the wild-type rat whole retina by RT–PCR (Figure 3). The whole-retina RT–PCR indicated that the retina has V1a and V1b AVP receptors. In regard to V2 receptors, our primer pairs could detect both the V2 receptor gene (301 base pairs) and opposite DNA strand of the *ARHGAP4* gene, a Rho GTPase-activating protein 4 gene (462 bp) [24] that overlaps the V2 receptor gene in the rat (Figure 3, V2/*ARHGAP4* lane). However, a V2 band could be distinguished by its size from an *ARHGAP4* band. In addition, by cloning and sequencing the RT–PCR product, we concluded that the V2/*ARHGAP4* band in Figure 3 was a band of *ARHGAP4*, not the V2 receptor. In contrast, no AVP receptor was detected by RT–PCR in dissociated AVP-eGFP-positive cells (Figure 3B, data from the same dissociated AVP-eGFP-positive cells shown in Figure 2B). Taken together, we concluded that the retina has an AVP-synthesizing and -receiving mechanism that can operate in a paracrine, not autocrine, manner through V1a and V1b AVP receptors.

### Arginine vasopressin secretion was not detected by stimulation with high potassium solution

**Figure 4:**
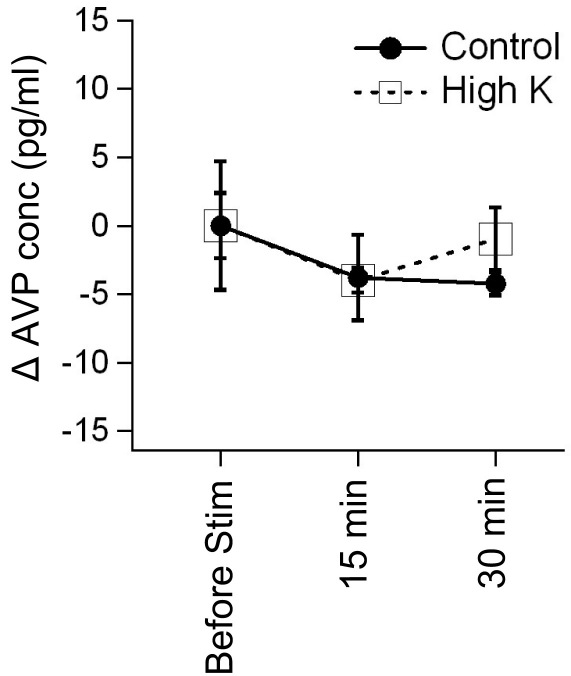
Arginine vasopressin secretion was not detected by stimulation with high potassium solution. The arginine vasopressin (AVP) concentration was assayed at 0 min (before stimulation), 15 min, and 30 min after the retina pieces were incubated in the control solution or the high potassium (50 mM) solution (see Methods). The concentration of AVP at each time was estimated under the condition that the concentration was set to 0 in the solution before incubation of the retina. The delta (Δ) AVP concentration shown here is defined as the difference between the concentration of AVP each time and the concentration before the incubation. No significant differences were found (one-way ANOVA with Tukey’s post hoc test). Error bars indicate standard error of the mean (SEM).

The remaining question is whether endogenous AVP is actually secreted in the retina under physiological conditions. To answer this question, we investigated endogenous AVP release from the retina by stimulating isolated wild-type retinas with high potassium solution, which has been shown to stimulate AVP release in the hypothalamus of the brain [25]. Isolated retinas were rinsed once in the control solution, and then each retina was incubated for 15 or 30 min in the control solution or the high potassium (50 mM) solution. The AVP concentration was assayed each time. Unfortunately, AVP levels were too low to detect AVP secretion from the retina by stimulation with the high potassium solution (Figure 4). We repeated the experiment using a batch of dissociated retinal cells instead of whole retinas, but we did not detect AVP secretion (data not shown).

## Discussion

We examined the possibility that endogenous AVP is synthesized in a certain type of retinal cell. Using AVP-eGFP transgenic rats, (1) immunohistochemistry showed that endogenous AVP positive cells are present in both the GCL and INL, (2) RT–PCR showed that endogenous *AVP* mRNA is present in GFP-positive cells, and (3) whole-retina RT–PCR showed that AVP receptors V1a and V1b are present in the retina. These findings suggest that the retina has an intrinsic AVP-synthesizing and -receiving mechanism that can operate in a paracrine manner.

We did not, however, detect AVP secretion from the retina. The endogenous AVP expression level in the wild-type retina was probably too low to be detected. In the present results, we detected only a faint band of endogenous *AVP* mRNA by RT–PCR (Figure 2). The band of endogenous *AVP* mRNA was fainter than that of the transgene of AVP-eGFP, even after 45 cycles of RT–PCR. It is possible that the expression level of endogenous AVP is very low. However, as no attempt at quantitative PCR has been made, the result does not necessarily mean that the AVP expression level is low. Moreover, the endogenous AVP expression level might be dynamically regulated by intracellular machinery or environmental factors in the retina. In fact, in the hypothalamic AVP neurons, AVP expression was regulated by various kinds of acute and chronic stresses such as osmotic changes or inflammatory/nociceptive stresses [26].

The results of the present study have not clarified the functional roles of endogenous AVP in the retina. The eye is a rather closed environment, and in addition to its vascular effects, AVP from retinal cells might therefore act within the retina in a paracrine manner through V1a and V1b receptors. Retinal endogenous AVP might have functional roles under pathological conditions such as high intraocular pressure (IOP). Injection of AVP into the anterior chamber or the vitreous chamber caused significant reduction in IOP and pupil size, probably due to stimulation of V1 receptors inside the eye [13,15]. It is therefore possible that endogenous AVP is expressed at a high level under pathological conditions such as high IOP to regulate the pressure.
